# Contribution of Cation-π Interactions in Iminium Catalysis

**DOI:** 10.3390/molecules17022161

**Published:** 2012-02-21

**Authors:** Yukie Mori, Shinji Yamada

**Affiliations:** Department of Chemistry, Ochanomizu University, Otsuka, Bunkyo-ku, Tokyo, 112-8610, Japan; Email: mori.yukie@ocha.ac.jp

**Keywords:** cation-π interaction, iminium-π interaction, iminium cation, imidazolidinone organocatalyst, *ab initio* calculation

## Abstract

*Ab initio* calculations were carried out for a benzyl-substituted iminium cation derived from (*E*)-crotonaldehyde and a chiral imidazolidinone that was developed as an organocatalyst by MacMillan *et al.* At the MP2 level of theory it is predicted that the phenyl group is close to the iminium moiety in the most stable conformer, suggesting that the cation-π interaction contributes to the stabilization of this conformer. Energy decomposition analyses on model systems indicate that the electrostatic and polarization terms make significant contribution to the attractive interactions between the benzene ring and the iminium cation.

## 1. Introduction

In organocatalytic reactions, non-covalent interactions as well as steric hindrance are crucial for the achievement of high selectivity as they affect the conformations of the reaction intermediates and/or transition states. Such interactions, for example, π-π, CH-π and cation-π interactions, are rather weak and therefore, it may be difficult to predict the dominant conformers for the species involving the key steps. Information on the factors that control the conformation of the reactive species will be valuable in the design of new organocatalysts with high reactivity and selectivity.

**Scheme 1:**
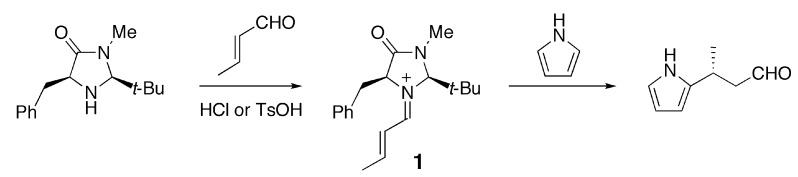
Iminium intermediate **1** derived from (*E*)-crotonaldehyde and the imidazolidinone developed by MacMillan *et al* [2].

In the present paper, we focus on a benzyl-substituted iminium cation **1** [1] and demonstrate that the cation-π interaction has a significant influence on the conformational preference for this reaction intermediate. Iminium cation **1** is formed from (*E*)-crotonaldehyde and a chiral imidazolidinone, which was developed by MacMillan and coworkers as an enantioselective organocatalyst (Scheme 1) [2].

**Figure 1:**
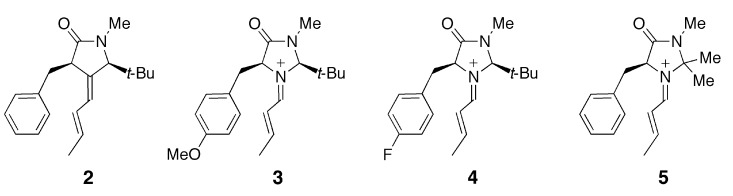
Structural formulas of the species related to the iminium intermediate **1**.

This intermediate undergoes various types of C-C bond formation reactions including Friedel-Crafts alkylation [2,3,4], Diels-Alder cycloaddition [1,5] and 1,3-dipolar additions [6]. MacMillan *et al.* attempted to explain the observed enantioselectivity of the products based on the conformation of the iminium intermediate predicted by MM3 calculations [2,3]. Houk *et al.* later estimated the relative energies of plausible conformers for the iminium intermediate and transition state in the Friedel-Crafts alkylation of pyrrole or *N*-methylindole by DFT calculations [7,8]. They suggested that the *E*/*Z* configuration of the C=N bond and the conformation of the benzyl group significantly affect the stereoselectivity of the product. Although the results of the calculations by Houk *et al.* can account for the experimental observations, they do not explain the origin of the conformational preference. One of our group recently proposed the cation-π interaction as one of the factors determining the conformational preference of **1** [9]. According to calculations at the B3LYP/6-31G(d) level, the most stable conformation of **1** displays a short distance between the iminium and phenyl moieties. In the case of the carbon-analogue **2**, such a conformer is slightly less stable than another conformer in which the phenyl group is more distant from the iminium moiety. Density functional theory, particularly the B3LYP hybrid functional, has been widely used for exploring organic reaction mechanisms [10]. In general, DFT methods may be inadequate for evaluating the magnitudes of non-covalent interactions such as dispersion interactions and charge-transfer interactions. In the present study, we have carried out quantum chemical calculations at the MP2 level of theory for different conformers of iminium **1** and its related species **2**–**4** (Figure 1). 

Furthermore, energy decomposition analyses have been applied to interacting pair models consisting of a truncated iminium cation and benzene in order to characterize the non-covalent interactions between the phenyl and iminium moieties. The results of these calculations provide a better understanding of cation-π interactions and suggest that such interactions may be utilized to improve the selectivities of organocatalysts through stabilization of the desired conformers.

## 2. Results and Discussion

### 2.1. Conformational Preferences in Iminium Intermediate ***1*** and Related Species ***2–5***

**Figure 2:**
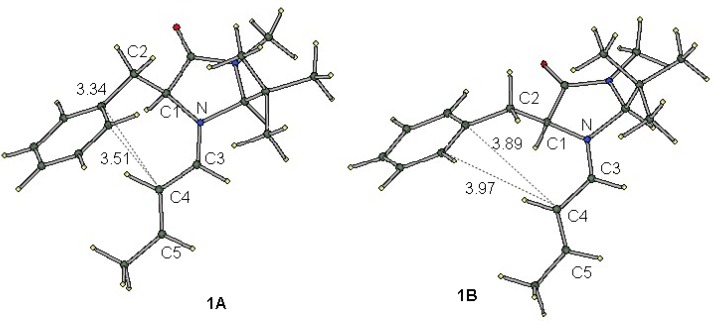
Optimized structures of conformers **1A** (Left) and **1B** (Right) with atom numbering. The figures indicate the interatomic distances in Å.

Figure 2 shows the B3LYP-optimized structure of the most stable conformers for iminium intermediate **1**. These conformers are different from each other in the orientation of the phenyl group; the dihedral angles of N−C1−C2−Ph are −72.8° and −134.5° for **1A** and **1B**, respectively. As a result, the distance between the phenyl and iminium moieties is shorter in **1A** than in **1B**. Short contacts of less than 3.6 Å are seen between the iminium α-carbon (C4) and the phenyl group in conformer **1A**. The interatomic distances C4−C*_ipso_* and C4−C*_ortho_* are 3.34 and 3.51 Å, respectively, while the corresponding distances in **1B** are much longer (3.89 and 3.97 Å, see Table 1). As is seen in Figure 2, the N=C3−C4=C5 π-system and the benzene ring are not parallel in either **1A** or **1B**.

The relative electronic energies of these conformers for iminium intermediate **1** and the related species **2**–**5** are summarized in Table 1. As was previously predicted by the DFT calculations [9], conformer **1A** is more stable than **1B**. For all the iminium cations **1**, **3**, **4** and **5**, conformer A is more stable than conformer B according to either DFT or MP2 calculations. On the other hand, the two conformers of the carbon analogue, **2A** and **2B**, were predicted to have almost the same energy. The difference in the conformational preference between the iminium cations and the non-charged carbon analogue suggests that the cation-π interaction plays an important role in the stabilization of conformer A. 

**Figure 3:**
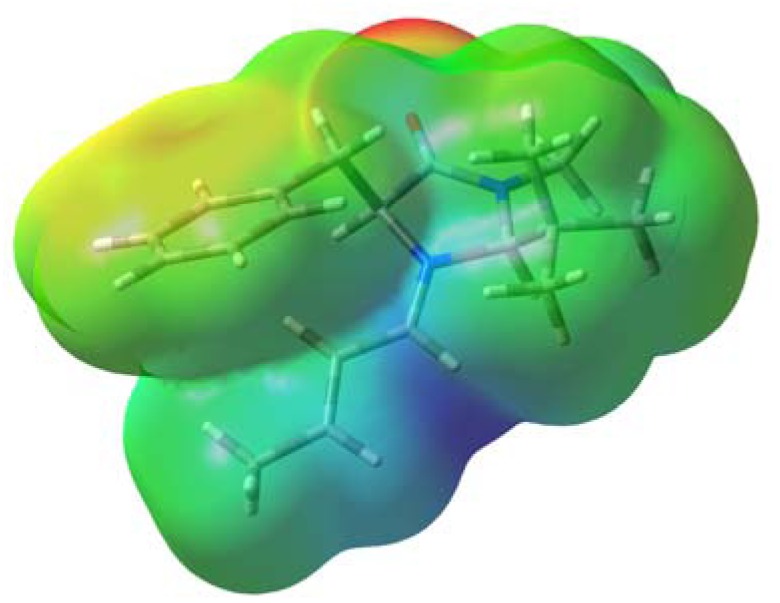
Electrostatic potential map for conformer **1A** plotted onto an isodensity surface (0.004 e au^−3^) obtained from the density calculated at the B3LYP/6-31G(d) level. The colour code corresponds to the charge from 0.03 (red) to 0.16 (dark blue).

Figure 3 shows the electrostatic potential for conformer 1**A**. The iminium moiety possesses high positive charge while the phenyl group is only slightly positive. Thus, the polarisable π-electrons in the phenyl group are likely to be attracted by the positive charge in the iminium moiety. It is expected that such an interaction would be enhanced with increases in the electron density of the π-system. We carried out calculation for the corresponding *p*-methoxy- and *p*-fluoro derivatives, **3** and **4**, at the same level of theory in order to examine the effects of the electron-donating and electron-withdrawing substituents. The predicted energy difference between conformer A and B increased in the order **4** (X = F) < **1** (X = H) < **3** (X = OMe) as shown in Table 1. This result supports the assumption that the cation-π interaction contributes to the predominance of conformer A over B.

**Table 1:** Relative electronic energies (kcal/mol) and non-bonded interatomic distances (Å) for conformers A and B of **1**–**5**.

	B3LYP	MP2	HF	C4 ^a^ -- C *_ipso_*	C4 ^a^ -- C *_ortho_*
**1A**	0.00 ^b^	0.00	0.00	3.34	3.51
**1B**	0.91 ^b^	2.00	0.32	3.89	3.97
**2A**	0.00 ^b^	0.00	0.00	3.47	3.57
**2B**	−0.38 ^b^	0.86	−0.64	4.11	4.23
**3A**	0.00	0.00	0.00	3.33	3.56
**3B**	1.09	2.33	0.53	3.82	3.93
**4A**	0.00	0.00	0.00	3.35	3.52
**4B**	0.74	1.75	0.00	3.89	3.99
**5A** ^c^	0.00	−	−	3.29	3.48
**5B** ^c^	1.03	−	−	3.77	3.83

^a^ C4 is the α-carbon in the iminium moiety, C3=N, see Figure 2; ^b^ Reference [9]; ^c^ Reference [8].

It should be noted that the cation-π interaction is not the only factor controlling the relative stabilities of the conformers for this type of iminium intermediates. Houk *et al.* predicted that the most stable conformer for a similar iminium cation **5** is not **5A**, but rather a different conformer in which the benzene ring faces one of the methyl groups at position C2 in the imidazolidine ring [7,8]. According to their calculations at the B3LYP/6-311+G(d,p)//B3LYP/6-31G(d) level, the electronic energy of conformer **5A** is only 0.2 kcal/mol higher than that of the most stable conformer. The energy difference between **5A** and **5B** (1.03 kcal/mol) is quite similar to that between **1A** and **1B** (0.91 kcal/mol) at the B3LYP/6-31G(d) level. It is likely that the cation-π interaction stabilizes conformer **5A** relative to **5B** as in the case of iminium **1**. Thus, a subtle balance of the non-bonding interactions governs the relative stabilities of conformers for the iminium intermediates.

### 2.2. Origin of Interaction between the Iminium and Phenyl Moieties

**Figure 4:**
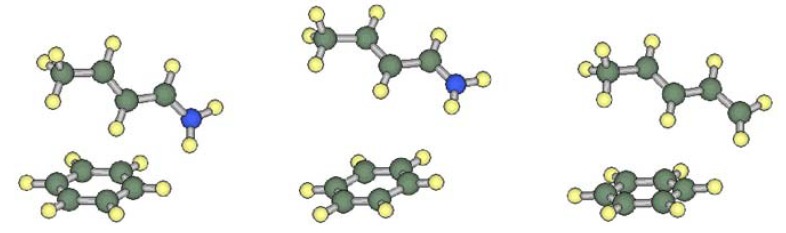
Interacting pair models **M1A** (left), **M1B** (middle) and **M2A** (right).

In order to characterize the interaction between the iminium and phenyl moieties in **1A**, we calculated the interaction energy for a hypothetical interacting pair model **M1A**, which consists of a (*E*)-but-2-eniminium cation and a benzene molecule (see Figure 4). The mutual orientation of the components was fixed to be the same as that in **1A**. The models corresponding to **1B**, **2A**, **3A** and **4A** were also examined for comparison. The interaction energies corrected for basis set superposition error (BSSE) are listed in Table 2. The MP2 calculation predicted that significant attractive interactions work between the iminium cation and the arene in models **M1A**, **M3A** and **M4A**. In model **M1B**, this interaction is weaker as the separation distance of the components is larger. In contrast, the interaction energy between the diene and benzene in **M2A** is close to zero.

To clarify the origin of the attractive interactions, the total interaction energies, E(MP2), were decomposed into the individual contributions from several terms. The correlation energies, defined as E(correlation) = E(MP2)−E(HF), are similar to each other for **M1A**, **M2A**, **M3A** and **M4A** as shown in Table 2. On the other hand, the stabilization energy within the Hartree-Fock approximation depends on the electronic nature of the components, and the order is **M2A** < **M4A** < **M1A** < **M3A**. That is, the attractive interaction is the largest between the iminium and the electron-rich arene. The E(HF) term was further decomposed into the electrostatic, polarization, charge-transfer, exchange-repulsion, and higher-order coupling terms by using the Morokuma-Kitaura energy decomposition scheme [11]. In the case of **M1A**, the magnitude of summed E(electrostatic) and E(polarization) is larger than the magnitude of E(correlation). The charge-transfer interaction also contributes to the stabilization of **M1A** although only small fractional charge (0.08*e*) is transferred from the iminium to benzene. The magnitude of any type of interaction is attenuated in **M1B** due to the larger separation between the two components. 

Tsuzuki and Yamada investigated the cation-π interactions between benzene and pyridinium (or *N*-methylpyridinium) cation with high-level ab initio calculations up to the CCSD(T) level at the basis set limit [12]. According to their calculations, there are local minima in which the C-H bond is directed toward the benzene plane (edge-to-face complex) with total stabilization energies of ca. −8 kcal/mol. They applied distributed multipole analyses with MP2/cc-pVTZ wavefunctions and revealed that the contribution from the electrostatic and induction terms is larger than the magnitude of the correlation energies. For the present models **M1-M4**, the geometries were not optimized but fixed at positions identical to those in the real systems **1**–**4**. At such non-optimal geometries, the total stabilization energies of **M1A** and **M3A** are rather large [13]. The dominance of electrostatic and polarization terms is also similar to the trends observed for the above benzene-pyridinium complexes, although we used an easier energy decomposition scheme with Hartree-Fock wavefunctions.

In contrast to the iminium-benzene pairs, the correlation effect dominates over the electrostatic or polarization effects for the chargeless diene-benzene pair, **M2A**. In the real systems **1A**, **1B**, **3A** and **4A**, the electrostatic interaction will be somewhat diminished since the sum of the atomic charges in the iminium moiety amounts to 0.6−0.7 rather than unity. However, the significant difference between **M1A** and **M2A** clearly indicates that the attractive interaction between the iminium and phenyl moieties in **1A** can be understood in terms of the cation-π interaction, in which the electrostatic attraction makes a sizable contribution. Thus, the cation-π interaction appears to be one of the factors that controlling conformational preference in the iminium intermediates for organocatalytic systems.

**Table 2:** Interaction energies (kcal/mol) and transferred fractional charges for the interacting pair models.

	M1A	M1B	M2A	M3A	M4A
E(MP2) ^a^	−5.32	−3.60	−0.30	−6.58	−3.44
E(HF) ^a^	0.08	−1.08	4.81	−0.47	2.32
E(correlation) ^b^	−5.40	−2.52	−5.10	−6.10	−5.76
E(electrostatic)	−4.34	−0.58	−2.09	−	−
E(polarization)	−3.05	−2.50	−0.35	−	−
E(charge transfer)	−2.10	−1.39	−1.41	−	−
E(exchange repulsion)	7.86	2.36	7.49	−	−
E(others)	0.91	0.54	0.30	−	−
BSSE	0.81	0.48	0.87	−	−
Transferred charge ^c^	0.08*e*	0.02	0.01	0.09	0.08

^a^ The basis set of 6-311G(d,p) was used. BSSE was corrected; ^b^ defined as E(MP2)−E(HF); ^c^ Based on the atomic charges calculated by electrostatic potential fitting at the MP2/6-311G(d,p) level.

## 3. Computational Details

The structures of iminium intermediate **1** and its carbon analogue **2** were taken from a previous work [9], in which they were optimized at the B3LYP/6-31G(d) level. The geometries of *p*-methoxy- or *p*-fluoro-substituted derivatives, **3** and **4** respectively, were optimized at the same level of theory. Single-point energy calculations were carried out at the MP2/6-31G(d,p) level to compare the electronic energies between conformers A and B for each species **1**–**4**.

The interacting pair models, which consisted of benzene and but-2-eniminium cation or 1,3-pentadiene, were constructed from the optimized structures of **1** or **2** by removing the atoms not belonging to the phenyl or N(or C)=CHCH=CHCH_3_ moiety followed by addition of terminating H-atoms at a distance of 1.087 Å (C_arom_-H), 1.00 Å (N-H) or 1.10 Å (C_olefin_-H). The interaction energies calculated at the MP2/6-311G(d,p) level for the fixed geometries as well as basis set superposition errors (BSSE) were corrected by the counterpoise method. The energy decompositionanalyses were performed based on the Morokuma-Kitaura scheme [11] for the HF/6-311G(d,p) reference wavefunctions. The atomic charges were obtained by electrostatic potential fitting using the Merz-Singh-Kollman scheme [14,15]. All the calculations were carried out with Gaussian03 [16] and GAMESS [17] program packages.

## 4. Conclusions

The present study revealed that the cation-π interaction manifests itself in benzyl-substituted iminium **1**, which is the key intermediate in the chiral imidazolidinone-catalyzed C-C bond formation developed by MacMillan *et al.* In the most stable conformer **1A**, the phenyl group is located close to the iminium moiety. The electrostatic and polarization effects contribute to the stabilization of this conformer together with the correlation interaction. These findings will provide an insight into the design of new organocatalysts with high reactivity and selectivity. As demonstrated by the present study, quantum chemical calculations provide valuable information on non-covalent interactions affecting the conformation of key intermediates in organocatalytic reactions.

## Supplementary Materials

Supplementary File 1
