# Supplementary material for: Contribution of Cation-π Interactions in Iminium Catalysis
Source: Molecules. 2012 Feb 21;17(2):2161–8. doi: 10.3390/molecules17022161 (PMC6268170; doi:10.3390/molecules17022161)
Supplement: Supplementary File 1 [file molecules-17-02161-s001.pdf]

Supplementary Information

Contribution of Cation-π Interactions in Iminium Catalysis

Yukie Mori and Shinji Yamada*

Department of Chemistry, Ochanomizu University, Otsuka, Bunkyo-ku, Tokyo, 112-8610, Japan;
E-Mail: yamada.shinji@ocha.ac.jp

1. Cartesian coordinates for optimized structure of **1A** **S2**
2. Cartesian coordinates for optimized structure of **1B** **S3**
3. Cartesian coordinates for optimized structure of **2A** **S4**
4. Cartesian coordinates for optimized structure of **2B** **S5**
5. Cartesian coordinates for optimized structure of **3A** **S6**
6. Cartesian coordinates for optimized structure of **3B** **S7**
7. Cartesian coordinates for optimized structure of **4A** **S8**
8. Cartesian coordinates for optimized structure of **4B** **S9**

Cartesian coordinates for optimized structure of **1A**

C	-0.330374	-0.993700	-0.728046
C	-1.694165	-1.619129	-1.035496
N	-2.667174	-0.678279	-0.822103
C	-2.182245	0.614434	-0.366900
H	-2.488102	1.397138	-1.075163
N	-0.692795	0.434013	-0.546688
H	0.294846	-1.074162	-1.621960
C	-4.047883	-0.944497	-1.219251
H	-4.677648	-1.206216	-0.364481
H	-4.027413	-1.793190	-1.905194
H	-4.467923	-0.070371	-1.725343
O	-1.854496	-2.761370	-1.410770
C	0.364486	-1.727652	0.447937
H	0.303208	-2.784976	0.163661
H	-0.235209	-1.614070	1.354098
C	1.801844	-1.331388	0.699949
C	4.473098	-0.561517	1.117393
C	2.826331	-1.834679	-0.116944
C	2.137243	-0.450744	1.736756
C	3.463638	-0.067423	1.945481
C	4.152440	-1.450791	0.087697
H	2.588717	-2.547083	-0.904523
H	1.359416	-0.076114	2.398934
H	3.709366	0.605538	2.762064
H	4.936294	-1.861449	-0.542435
H	5.506610	-0.273028	1.285514
C	0.080306	1.453539	-0.815038
H	-0.410417	2.423599	-0.796941
C	1.463997	1.423816	-1.152840
H	2.004558	0.483747	-1.165777
C	2.098312	2.589653	-1.441992
H	1.529738	3.520069	-1.395036
C	3.529389	2.709171	-1.818911
H	4.042247	3.393421	-1.129237
H	3.619797	3.164485	-2.815066
H	4.045240	1.746052	-1.815346
C	-2.635602	1.078090	1.064318
C	-4.138342	1.441882	0.990159
H	-4.432459	1.934286	1.922315
H	-4.780350	0.565984	0.875823
H	-4.352361	2.140483	0.172325
C	-1.869981	2.354693	1.468709
H	-0.796895	2.177814	1.599704
H	-2.250435	2.713997	2.430190
H	-2.013057	3.170945	0.748479
C	-2.422740	-0.005829	2.132708
H	-1.361955	-0.157358	2.357743
H	-2.857975	-0.965619	1.836703
H	-2.905085	0.301504	3.066094

Cartesian coordinates for optimized structure of **1B**

```
C  0.001994  -0.455687 -0.423299
C  -0.869438 -1.648410 -0.818731
N  -2.173740 -1.234771 -0.882897
C  -2.400689 0.139412  -0.463103
H  -2.926067 0.694645  -1.252186
N  -0.985547 0.648987  -0.429579
H  0.707559  -0.274258 -1.239174
C  -3.203973 -2.111452 -1.435892
H  -3.738387 -2.661336 -0.656230
H  -2.705220 -2.832836 -2.085835
H  -3.918091 -1.522573 -2.017549
O  -0.451203 -2.762441 -1.061458
C  0.793268  -0.650264 0.908433
H  0.370976  -1.509784 1.439845
H  0.632815  0.224613  1.547136
C  2.280380  -0.847557 0.681204
C  5.034091  -1.200107 0.268224
C  2.749519  -1.921647 -0.090143
C  3.204391  0.042560  1.243611
C  4.575504  -0.133389 1.040747
C  4.118320  -2.092747 -0.295172
H  2.041123  -2.624033 -0.522865
H  2.854290  0.864995  1.865206
H  5.281223  0.558344  1.492019
H  4.470802  -2.930254 -0.890332
H  6.099381  -1.340365 0.110193
C  -0.677332 1.905509  -0.591191
H  -1.513091 2.588859  -0.719995
C  0.647042  2.437436  -0.622533
H  1.498712  1.782856  -0.458652
C  0.832611  3.763513  -0.849357
H  -0.045332 4.394384  -0.998189
C  2.151628  4.441241  -0.913881
H  2.196502  5.246426  -0.167452
H  2.273941  4.931866  -1.889577
H  2.986086  3.754706  -0.752240
C  -3.190041 0.337627  0.882079
C  -4.660961 -0.071895 0.633048
H  -5.261837 0.195584  1.507895
H  -4.779153 -1.146584 0.481143
H  -5.092606 0.449684  -0.230042
C  -3.185471 1.826723  1.284260
H  -2.191006 2.189584  1.566785
H  -3.827060 1.963845  2.159974
H  -3.589461 2.470631  0.491784
C  -2.607459 -0.512848 2.022448
H  -1.605034 -0.185868 2.319057
H  -2.561866 -1.574074 1.757448
H  -3.247301 -0.424185 2.906127
```

Cartesian coordinates for optimized structure of **2A**

C	-0.314121	-0.942955	-0.747653
C	-1.656931	-1.606317	-1.064856
N	-2.666826	-0.708877	-0.828075
C	-2.219815	0.620768	-0.384086
H	-2.607599	1.372287	-1.090296
C	-0.698985	0.511769	-0.568654
H	0.327081	-1.067439	-1.628891
C	-4.022415	-1.032152	-1.238033
H	-4.654577	-1.340995	-0.397590
H	-3.964953	-1.864901	-1.942692
H	-4.484865	-0.165671	-1.722770
O	-1.809360	-2.753024	-1.462243
C	0.365683	-1.720384	0.423483
H	0.296570	-2.779900	0.150132
H	-0.224753	-1.590604	1.335077
C	1.808330	-1.354339	0.687070
C	4.500897	-0.648897	1.130236
C	2.836681	-1.920181	-0.081330
C	2.156371	-0.438854	1.689044
C	3.488646	-0.087163	1.909838
C	4.170324	-1.570775	0.134222
H	2.589040	-2.646595	-0.853007
H	1.375464	0.006473	2.300964
H	3.735430	0.625463	2.692752
H	4.951647	-2.025151	-0.469814
H	5.539214	-0.378125	1.302534
C	0.082777	1.586285	-0.794427
H	-0.383853	2.570725	-0.753926
C	1.496039	1.590173	-1.132469
H	2.007144	0.632596	-1.201968
C	2.195666	2.715028	-1.365511
H	1.684535	3.676501	-1.284642
C	3.649215	2.762130	-1.727661
H	4.218719	3.364657	-1.006455
H	3.801789	3.229235	-2.711049
H	4.088949	1.759677	-1.752658
C	-2.753985	1.061676	1.035824
C	-4.283717	1.280143	0.981377
H	-4.617591	1.781501	1.897472
H	-4.843347	0.343866	0.907746
H	-4.572673	1.917508	0.136200
C	-2.114215	2.409482	1.424844
H	-1.029481	2.328805	1.533278
H	-2.524228	2.753491	2.381916
H	-2.326407	3.188204	0.680589
C	-2.436624	0.021734	2.121794
H	-1.361099	-0.037500	2.318757
H	-2.789630	-0.975901	1.839336
H	-2.925769	0.296908	3.063765

Cartesian coordinates for optimized structure of **2B**

C	0.0715329	-0.4039802	-0.4610802
C	-0.7218034	-1.6554482	-0.8316995
N	-2.0547581	-1.3419601	-0.8756032
C	-2.3778065	0.0445003	-0.5009016
H	-2.9770320	0.4978337	-1.3057451
C	-0.9857742	0.6804230	-0.4683009
H	0.7856863	-0.2329081	-1.2752930
C	-2.9995535	-2.2990306	-1.4262843
H	-3.4903284	-2.9019387	-0.6535387
H	-2.4466295	-2.9769328	-2.0807815
H	-3.7685106	-1.7726379	-2.0006800
O	-0.2596256	-2.7638888	-1.0775276
C	0.8979863	-0.5754968	0.8594569
H	0.4570671	-1.3860221	1.4523900
H	0.7922106	0.3374823	1.4543560
C	2.3738312	-0.8504798	0.6382939
C	5.1188990	-1.3370253	0.2463477
C	2.8027017	-1.9546959	-0.1153149
C	3.3400507	0.0012756	1.1886637
C	4.7030799	-0.2376065	0.9966153
C	4.1633667	-2.1925072	-0.3083309
H	2.0604145	-2.6173178	-0.5530596
H	3.0223281	0.8593774	1.7779350
H	5.4360307	0.4362176	1.4333556
H	4.4790077	-3.0518140	-0.8949531
H	6.1782139	-1.5264604	0.0930689
C	-0.7438572	1.9987970	-0.5960078
H	-1.5890276	2.6797760	-0.6883623
C	0.5710982	2.6202926	-0.6374642
H	1.4428446	1.9721022	-0.5496607
C	0.7629518	3.9445129	-0.7757509
H	-0.1117409	4.5925174	-0.8574119
C	2.0989316	4.6208961	-0.8284103
H	2.2032035	5.3618458	-0.0233479
H	2.2309809	5.1686207	-1.7719688
H	2.9190861	3.9012579	-0.7362390
C	-3.2318542	0.1784642	0.8215758
C	-4.6336730	-0.4371078	0.6183587
H	-5.2710968	-0.1980454	1.4777814
H	-4.6081991	-1.5258870	0.5303906
H	-5.1258215	-0.0329247	-0.2755443
C	-3.4343710	1.6701583	1.1566170
H	-2.4958520	2.1642827	1.4203239
H	-4.1131802	1.7671166	2.0120026
H	-3.8848553	2.2168607	0.3177673
C	-2.5371863	-0.5116146	2.0064784
H	-1.5733788	-0.0428959	2.2312871
H	-2.3621919	-1.5758258	1.8129029
H	-3.1602244	-0.4348127	2.9052951

Cartesian coordinates for optimized structure of **3A**

C,-0.8828650677,-1.0187971835,-0.8878488246
C,-2.2357280513,-1.6580493602,-1.2083258531
N,-3.2217800013,-0.7267443484,-1.0093656271
C,-2.7527874097,0.5766533466,-0.5689176413
H,-3.0557124546,1.3451604788,-1.2940578041
N,-1.2592274504,0.406768015,-0.7223184811
H,-0.2470476504,-1.0995140917,-1.774385996
C,-4.5883725359,-1.0030657489,-1.4487437993
H,-5.2320787779,-1.3084337587,-0.6194485933
H,-4.5386818427,-1.8237598986,-2.1667494454
H,-5.0125199053,-0.1165870439,-1.9285485491
O,-2.3819919784,-2.8023894487,-1.5831449454
C,-0.1920031355,-1.7382487984,0.3012968375
H,-0.2418419601,-2.7976820762,0.0212843976
H,-0.8049302959,-1.6252304421,1.1985833253
C,1.2353794885,-1.3266964839,0.5678285168
C,3.908641401,-0.5377928261,1.0305079338
C,2.2776755086,-1.7772230942,-0.2641771923
C,1.5671619162,-0.4898507896,1.6369629915
C,2.8852451628,-0.0929892726,1.8782456704
C,3.5913977727,-1.3860458749,-0.0470624782
H,2.059315263,-2.4593679906,-1.0837310466
H,0.7882669351,-0.1476206334,2.315334893
H,3.1026088144,0.5430727111,2.7284320129
H,4.3988304462,-1.7465810498,-0.676501384
O,5.2187910351,-0.2199149247,1.1608430362
C,-0.4910008364,1.4323033543,-0.9840307598
H,-0.9933138798,2.3965506143,-0.9884334852
C,0.9004466125,1.4156209338,-1.2923009368
H,1.4563819491,0.4851500715,-1.2614625558
C,1.5213862555,2.581590044,-1.6085702202
H,0.9386137761,3.5043271166,-1.6018380483
C,2.956398913,2.7095035413,-1.9701078791
H,3.4506789566,3.4253561854,-1.2993405851
H,3.0531841471,3.1305356794,-2.9806578866
H,3.4851826676,1.7546275408,-1.9260101364
C,-3.2285566609,1.0601637453,0.84780908
C,-4.7437651383,1.3605387272,0.7574178174
H,-5.0686352864,1.8456409338,1.6830444367
H,-5.3441869576,0.4563154232,0.6396306291
H,-4.978876334,2.0425919982,-0.0687063036
C,-2.5163394782,2.3768871415,1.2223748665
H,-1.4408072676,2.2453579022,1.383283937
H,-2.9322817317,2.7538707508,2.1616536781
H,-2.6713970603,3.1610517306,0.4696374875
C,-2.978761831,0.012445559,1.9439782094
H,-1.9119987918,-0.1065759845,2.15930823
H,-3.3935058341,-0.9657711716,1.6803098121
H,-3.4586630232,0.3336730456,2.8739520471
C,5.6306826103,0.547398164,2.2892885404
H,5.1796851833,1.5478244509,2.2792273647
H,5.3775585404,0.0403699904,3.227996309
H,6.7139712728,0.6379420998,2.2053073973

Cartesian coordinates for optimized structure of **3B**

C,-0.64745602,-0.3965430848,-0.4268426825
C,-1.5119617404,-1.594242781,-0.8159359253
N,-2.8170229038,-1.1843363067,-0.900068576
C,-3.0506214014,0.1954010691,-0.5041762927
H,-3.5596800559,0.7404072529,-1.3110270913
N,-1.6358007528,0.7065450876,-0.4486636195
H,0.057766119,-0.2241670809,-1.2450824094
C,-3.8293813319,-2.0606433294,-1.485388379
H,-4.3868141871,-2.6130581707,-0.7239918688
H,-3.3099136689,-2.7806615017,-2.1204589873
H,-4.5255900738,-1.4714951932,-2.0884104869
O,-1.0872523433,-2.7087417817,-1.0446775655
C,0.1489526597,-0.5752401759,0.906077561
H,-0.2421949428,-1.4607178274,1.4194113655
H,-0.0537019078,0.2813735664,1.5576483247
C,1.6410667271,-0.7066152818,0.6822646264
C,4.4207403924,-0.9688548885,0.2868689511
C,2.1589721774,-1.7389993037,-0.122436578
C,2.5395917238,0.1807415436,1.2799633857
C,3.9207670068,0.0602915319,1.0941427058
C,3.5246431553,-1.8667993719,-0.3215498513
H,1.4806220073,-2.451162347,-0.587034095
H,2.167469,0.973510921,1.926864885
H,4.5874843919,0.7591799219,1.5857642657
H,3.9306736707,-2.6638702613,-0.9360332075
O,5.7334806662,-1.1859891743,0.0298161779
C,-1.322664738,1.9616075915,-0.6144524363
H,-2.1532919401,2.6483947238,-0.7562049264
C,0.0052974546,2.4862462908,-0.6398794226
H,0.8519899833,1.8253521765,-0.4724410176
C,0.199470469,3.8108272573,-0.8671567559
H,-0.6739949834,4.4480354243,-1.0154067407
C,1.5231282869,4.4803354249,-0.9344084281
H,1.5726128931,5.2905060839,-0.193933937
H,1.6489998048,4.9624215245,-1.9138531472
H,2.3524706995,3.7890056036,-0.7676300561
C,-3.8686095852,0.4113764742,0.8201932257
C,-5.3326604204,-0.0017056097,0.5434359037
H,-5.9502097353,0.2633456587,1.4072187414
H,-5.4427364536,-1.0767225392,0.3874828178
H,-5.7478148117,0.51922907,-0.3279340511
C,-3.8704611279,1.9055605624,1.2038464468
H,-2.8826966626,2.2656150577,1.5118478861
H,-4.538563918,2.0564079267,2.0572054902
H,-4.2449420335,2.5416204699,0.3907895113
C,-3.3038885532,-0.4216008129,1.9822666252
H,-2.2954379628,-0.1025469257,2.266362303
H,-3.2741525743,-1.4894769829,1.743903253
H,-3.9404716536,-0.2991319684,2.8643087172
C,6.7009361775,-0.3454602144,0.6496074833
H,6.5810854961,0.6998137374,0.3373275665
H,6.6433884062,-0.4102461457,1.7431912073
H,7.6711621148,-0.7137538916,0.3148191077

Cartesian coordinates for optimized structure of **4A**

```
C,-0.6624183104,-0.9826097437,-0.8115949143
C,-2.0281424829,-1.6016649434,-1.1220569247
N,-2.9989170058,-0.6619302796,-0.8952395499
C,-2.5092644114,0.6276196359,-0.4350082509
H,-2.8157031205,1.4144751817,-1.1381795397
N,-1.0201864786,0.4449790004,-0.6206523869
H,-0.0392100261,-1.058347261,-1.7073778282
C,-4.3795062839,-0.9212698735,-1.300545194
H,-5.0006822984,-1.2370920223,-0.4580426042
H,-4.3562052863,-1.7294618158,-2.0338505606
H,-4.8109510886,-0.0239643228,-1.7524688139
O,-2.1914935687,-2.7392320692,-1.5099041098
C,0.0305885514,-1.7283761305,0.3583927386
H,-0.0231069858,-2.7819898588,0.0596396844
H,-0.5762916407,-1.6302446521,1.2614840277
C,1.4626787142,-1.3270711293,0.6265845737
C,4.1019653242,-0.5505063473,1.0751329952
C,2.5024813889,-1.8249262223,-0.1754667103
C,1.7835677459,-0.4467330335,1.6694317717
C,3.1008849441,-0.05175296,1.90213471
C,3.8250511449,-1.4379454452,0.0378815491
H,2.2813779767,-2.5369821497,-0.967712869
H,0.9980519319,-0.0739447901,2.3226062227
H,3.3612034886,0.618543911,2.7145425291
H,4.6378262406,-1.8278931158,-0.565916536
F,5.37181732,-0.1709569257,1.2857804016
C,-0.248479268,1.4645678059,-0.895111368
H,-0.7391742086,2.4346734454,-0.8786118485
C,1.1340407136,1.4339860434,-1.2390400619
H,1.6751517334,0.4944517334,-1.2394413421
C,1.7653079738,2.5963056846,-1.5499130172
H,1.1958349287,3.5265537809,-1.5129994001
C,3.1931993099,2.7131883844,-1.9395756016
H,3.7138012033,3.3953087932,-1.2533265955
H,3.2750214108,3.1708487401,-2.9352526916
H,3.7075457141,1.7495647757,-1.9428368812
C,-2.9576223278,1.0855362047,0.999294649
C,-4.4630172024,1.4367792905,0.9283683689
H,-4.766657593,1.9055963047,1.8694454064
H,-5.0935977922,0.5561736299,0.7899551869
H,-4.6811547122,2.1488700773,0.123232898
C,-2.2000227974,2.367342883,1.4028959876
H,-1.1259317188,2.1990120175,1.5377802983
H,-2.5865816315,2.7265028712,2.3616139857
H,-2.3453269033,3.1801244613,0.6791708027
C,-2.7362892064,-0.0002824195,2.0641407758
H,-1.6736414529,-0.1579093502,2.274794178
H,-3.1807048066,-0.9577520413,1.7739325227
H,-3.2044401501,0.3084672463,3.0042513365
```

Cartesian coordinates for optimized structure of **4B**

C,-0.2179312559,-0.44499589,-0.424407212
C,-0.9958086672,-1.7011832712,-0.8150364515
N,-2.3271267863,-1.388315451,-0.8841379785
C,-2.6593272361,-0.031983373,-0.4761874786
H,-3.213939995,0.4782815035,-1.2755171452
N,-1.2855353374,0.5823777787,-0.4283600084
H,0.4656203472,-0.2140985641,-1.2467352885
C,-3.2780615479,-2.3349617882,-1.4643286878
H,-3.7775207424,-2.937186048,-0.7005522465
H,-2.7154890723,-3.0051809228,-2.1168919326
H,-4.0282273772,-1.7951307545,-2.0482550067
O,-0.4921335263,-2.7803583346,-1.0544815175
C,0.597282253,-0.5755705247,0.90046824
H,0.2429102195,-1.4617326575,1.4379700137
H,0.3777155607,0.287197244,1.538007118
C,2.0922006056,-0.6649333318,0.6610812106
C,4.8379847659,-0.8286806153,0.236581354
C,2.6304223825,-1.6920421965,-0.1313752376
C,2.9609412223,0.2714463991,1.2372658425
C,4.3395648428,0.1965068019,1.0317436354
C,4.0034045664,-1.7767297135,-0.3497585538
H,1.9696225437,-2.4323863191,-0.5756196941
H,2.5643545637,1.0593738753,1.874379213
H,5.0233930087,0.908725073,1.4811786473
H,4.4337415041,-2.566617075,-0.9562301408
F,6.1616689748,-0.9072446834,0.0283528479
C,-1.0683978651,1.8579233759,-0.5933223728
H,-1.9499312373,2.4809265398,-0.7216151864
C,0.2147192614,2.4826834669,-0.6353650755
H,1.1127737408,1.889226637,-0.4862757815
C,0.3032369443,3.8192062368,-0.861385714
H,-0.6193249242,4.3868715711,-0.9938962635
C,1.569018044,4.5897871773,-0.9473434441
H,1.5675830699,5.3979152242,-0.2028950336
H,1.6386109994,5.0856638126,-1.9255570825
H,2.4535754714,3.9658815859,-0.7996159982
C,-3.4791574805,0.1150584957,0.8561829538
C,-4.9076913868,-0.4148195758,0.5933768914
H,-5.5362916755,-0.200256561,1.4631695317
H,-4.9312470539,-1.4952599014,0.4383817029
H,-5.3723397581,0.0703569643,-0.2736257153
C,-3.5969556645,1.6040248099,1.2418808077
H,-2.6386445588,2.0419600674,1.5422414356
H,-4.2671754395,1.7001424864,2.1014212276
H,-4.0285573733,2.2089822346,0.4334288355
C,-2.8370136125,-0.6706718079,2.0109070818
H,-1.8560637358,-0.2691804358,2.2870318864
H,-2.7218237419,-1.732100564,1.7693075928
H,-3.4732230712,-0.6031305356,2.8990609912
